# Neuronal patterning of the tubular collar cord is highly conserved among enteropneusts but dissimilar to the chordate neural tube

**DOI:** 10.1038/s41598-017-07052-8

**Published:** 2017-08-01

**Authors:** Sabrina Kaul-Strehlow, Makoto Urata, Daniela Praher, Andreas Wanninger

**Affiliations:** 1 Department for Integrative Zoology, University of Vienna, Althanstr. 14, 1090 Vienna, Austria; 2 Research Center for Marine Biology, Tohoku University, Asamushi, Aomori, Aomori, 039-3501 Japan; 3 Department for Molecular Evolution and Development, University of Vienna, Althanstr. 14, 1090 Vienna, Austria; 4 Noto Marine Laboratory, Division of Marine Environmental Studies, Institute of Nature and Environmental Technology, Kanazawa University, Ogi, Noto-cho, Ishikawa, 927-0553 Japan

## Abstract

A tubular nervous system is present in the deuterostome groups Chordata (cephalochordates, tunicates, vertebrates) and in the non-chordate Enteropneusta. However, the worm-shaped enteropneusts possess a less complex nervous system featuring only a short hollow neural tube, whereby homology to its chordate counterpart remains elusive. Since the majority of data on enteropneusts stem from the harrimaniid *Saccoglossus kowalevskii*, putative interspecific variations remain undetected resulting in an unreliable ground pattern that impedes homology assessments. In order to complement the missing data from another enteropneust family, we investigated expression of key neuronal patterning genes in the ptychoderid *Balanoglossus misakiensis*. The collar cord of *B. misakiensis* shows anterior *Six3/6* and posterior *Otx* + *Engrailed* expression, in a region corresponding to the chordate brain. Neuronal *Nk2.1/Nk2.2* expression is absent. Interestingly, we found median *Dlx* and lateral *Pax6* expression domains, i.e., a condition that is reversed compared to chordates. Comparative analyses reveal that adult nervous system patterning is highly conserved among the enteropneust families Harrimaniidae, Spengelidae and Ptychoderidae. *BmiDlx* and *BmiPax6* have no corresponding expression domains in the chordate brain, which may be indicative of independent acquisition of a tubular nervous system in Enteropneusta and Chordata.

## Introduction

**Figure 1:**
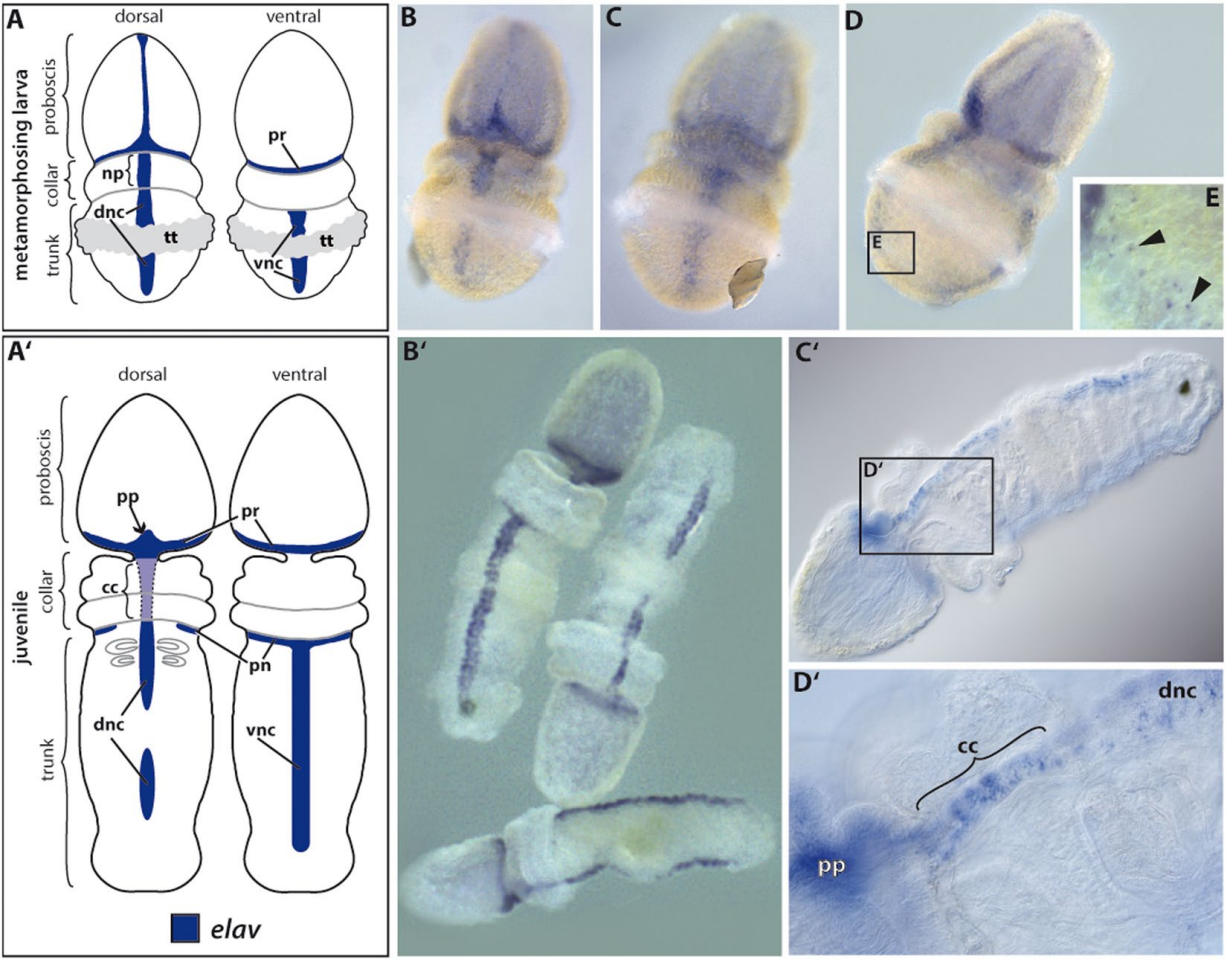
Establishment of the adult nervous system. Gene expression of *BmiElav* in the metamorphosing larva and juvenile of *B. misakiensis*. (**A**–**E**) Metamorphosing larva. (A’–D’) Juvenile. (**A**) Schematic illustration of *BmiElav* expression. *BmiElav* is expressed in the proboscis nerve ring, the developing dorsal nerve cord including the neural plate in the collar (**B,D**) and in the ventral nerve cord (**C,D**). Note that the expression is interrupted at the level of the telotroch. E Detail of the lateral trunk showing scattered neurons (arrowheads). (A’) Schematic illustration of *BmiElav* expression in juveniles. Note that the collar cord is neurulated. B’ Surface view from ventral, dorsal and lateral right (from top to bottom) showing strong expression in the proboscis nerve ring, proboscis plexus, and in the dorsal as well as ventral nerve cord. *BmiElav* expression is discontinuous in the middle of the dorsal nerve cord in the trunk region. (C’) Micrograph of cleared juvenile. (D’) Detail showing *Elav*+ cells in the subepidermal collar cord. cc = collar cord, dnc = dorsal nerve cord, np = neural plate, pn = peribranchial nerve ring, pr = proboscis nerve ring, pp = proboscis plexus, tt = telotroch, vnc = ventral nerve cord. (**B**) dorsal view. (**C**) ventral view. (**D**) view from lateral right.

The evolution of the nervous system in Bilateria and Deuterostomia in particular has been hotly debated for decades^1–6^. In this debate, enteropneust hemichordates (or acorn worms) have occupied a pivotal role. Enteropneusts and echinoderms are two groups of non-chordate deuterostomes, distantly related to vertebrates^7^. In contrast to echinoderms that feature a highly derived body organization (pentamery, oral-aboral axis), the worm-shaped enteropneusts have retained putative ancestral bilaterian features such as bilateral symmetry, nephridia, coelomic cavities and a biphasic life style^8^. Enteropneusts share some characteristics with chordates, such as gill slits and, at least partly, a tubular nervous system. For these reasons, enteropneusts are ideal candidates to unravel nervous system evolution in Deuterostomia. The majority of enteropneust species belong to one of the three main families Harrimaniidae (e.g. *Saccoglossus kowalevskii*), Spengelidae (e.g. *Schizocardium californicum*) and Ptychoderidae (e.g. *Balanoglossus misakiensis*, *Ptychodera flava*)^9^. Harrimaniid species develop directly into the juvenile worm, whereas spengelid and ptychoderid enteropneusts develop indirectly via a specific larval type, the tornaria. Morphologically, the nervous system of enteropneusts is a basiepidermal plexus with additional condensed regions^10^. These comprise the proboscis stem, proboscis nerve ring, a dorsal nerve cord along the collar and trunk region, and a ventral nerve cord in the trunk connected to the dorsal nerve cord by a prebranchial nerve ring (Fig. 1A’)^10, 11^. The dorsal nerve cord within the collar region, the ‘collar cord’, is a subepidermal tubular nerve cord that is often thought to be reminiscent of the chordate neural tube and, like the latter, forms by neurulation^12, 13^. The collar cord is subdivided into a dorsal sheath of different neuronal cell types surrounding a central neural canal and a ventral neuropil^13, 14^. Although these morphological features would support homology of the chordate neural tube and the collar cord of enteropneusts, it remains unclear as to which part of the chordate neural tube the collar cord might correspond. Moreover, the results from gene expression analyses are somewhat contradictory. The nervous system of many bilaterians is patterned similarly from anterior to posterior by a number of specific transcription factors (see ref. 3 for review). For instance, *Six3/6, Otx* and E*ngrailed* regionalize parts of the brain in bilaterians, while *Hox* genes pattern the postcerebral nerve cord^3^. Anteroposterior patterning of these transcription factors has been studied in the enteropneust *S. kowalevskii* and is similar to that in chordates^3, 15, 16^, yet the expression domains in *S. kowalevskii* are circumferential in the entire ectoderm and not restricted to the neuroectoderm as in chordates^15, 16^. The spengelid *S. californicum* exhibits similar expression domains of these transcription factors^17^. To complicate things further, Miyamoto and Wada^18^ showed that genes specifying the chordate neural plate border (e.g., *SoxE*, and *Bmp2/4*) have corresponding expression domains in the neural plate of the collar cord of the enteropneust *Balanoglossus simodensis* ^18^. Concluding so far, no unequivocal homology statement can be made at present concerning the collar cord and the chordate neural tube.

Most of the gene expression data available for enteropneusts have been obtained from *S. kowalevskii*, a harrimaniid species with direct development. This data has been supplemented recently by a body patterning study of the spengelid *S. californicum* ^17^. However, a reliable ground pattern of neuronal patterning for Enteropneusta can only be reconstructed if data from members of many different enteropneust families are compared. Accordingly, a comparable developmental study of neuronal patterning in a ptychoderid enteropneust is of prime importance.

**Figure 2:**
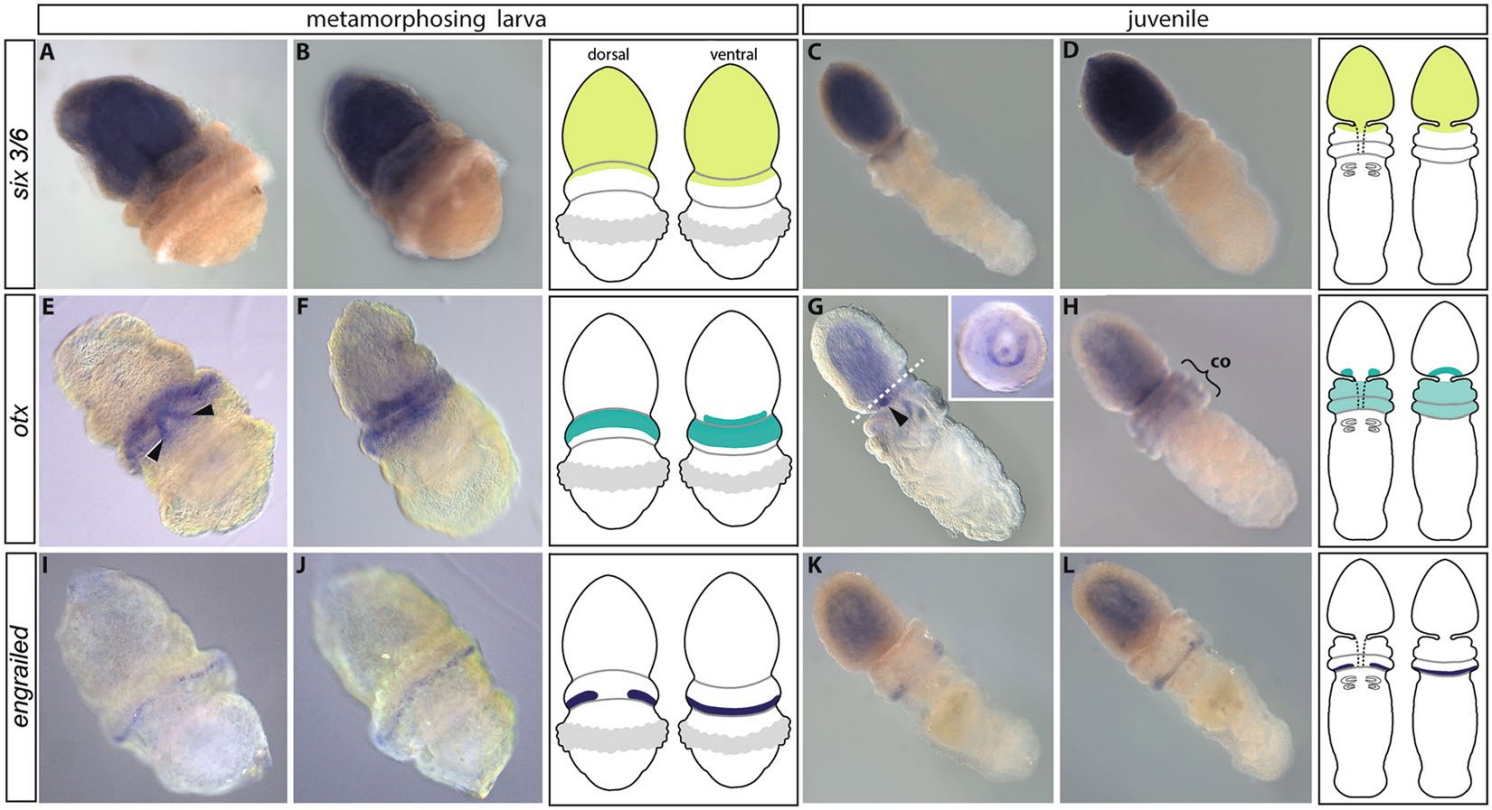
Anteroposterior patterning genes allocate the collar cord of *B. misakiensis* to the chordate brain region. Anterior is to the top left. (**A**–**D)** *BmiSix3/6* is expressed throughout the ectoderm of the proboscis region and the anterior collar. (**E**–**H)** *BmiOtx* is expressed circumferentially in the posterior proboscis ectoderm and in the ectoderm of the collar region. (**E)** Dorsal view showing an additional domain in the pharyngeal endoderm (arrowheads). (**G)** *BmiOtx* is strongly expressed in the preoral ciliary organ (arrowhead). Section plane of inset indicated by dashed line. Inset shows *Otx* expression in the ciliary organ of the proboscis in a cross section of the posterior proboscis. (**I**–**L)** *BmiEn* is expressed in a narrow ring in the ectoderm of the posterior end of the collar region with an interruption on the dorsal side. co = collar. (**A**,**C**,**E**,**G**,**I**,**K**): dorsal views. (**B**,**D**,**F**,**H**,**J**,**L**): ventral views.

Here, we studied the expression domains of neuronal patterning genes in the indirectly developing ptychoderid *Balanoglossus misakiensis*, in order to provide the missing data. We focused on the expression patterns in the developing collar cord of anteroposterior (*Six3/6, Otx*, and *Engrailed*) as well as putative mediolateral patterning genes (*Pax6, Dlx, Nk2.1, Nk2.2*). The latter have been reported to form abutting domains of *Nk* and *Pax* genes in the annelid ventral nerve cord and in the vertebrate dorsal neural tube^2, 19^. In each of these progenitor domains specific neuronal cell types are formed (see Fig. 7 in ref. 19). For instance, serotonin-positive (+) neurons are exclusively restricted to the median *Nk2.1* domain in the brain and to the median *Nk2.2* in the spinal cord (see Figs 2 + 3 in ref. 2). *Pax6* forms two bilaterally symmetric, intermediate progenitor domains and *Dlx* two lateral domains. Given the presence of a corresponding spatial organization of the vertebrate neural tube and the nerve cord in annelids, a similarly patterned nervous system has been proposed in the last common ancestor of Bilateria^3^. Therefore, we assess the presence of putative mediolateral patterning in the collar cord of *B. misakiensis*. The adult nervous system of *B. misakiensis*, including the collar cord, becomes morphologically distinct in early settled juveniles, indicating that neurogenic patterning of the collar cord starts in metamorphosing larvae^20^. In contrast, the larval nervous system (apical organ and neurite bundles of the ciliary bands) is independent of the adult nervous system and degrades during metamorphosis and settlement^9, 18, 21^. Therefore, we did not study the larval stages, but rather focus on the expression patterns in metamorphosing animals and early settled juveniles.

This study describes the first gene expression data for the ptychoderid *B. misakiensis*, and will enable the establishment of a reliable ground pattern for Enteropneusta. The 2^nd^ objective of this study is then to compare the collar cord with the chordate neural tube, in order to elucidate the evolution of tubular nervous systems in Deuterostomia.

## Results

### Neuronal differentiation of the adult nervous system

In order to obtain an overview of the developing adult nervous system of *Balanoglossus misakiensis*, we first examined the expression of *Elav*, an RNA-binding protein that marks differentiating neurons^22–24^. *BmiElav* is expressed in the epidermis of the metamorphosing larva (Agassiz stage) of *B. misakiensis* as a stripe along the entire dorsal midline (except at the level of the telotroch) and extends circumferentially to the posterior base of the proboscis (Fig. 1A,B,D). In addition, *BmiElav* expression runs along the ventral midline of the trunk region with a gap in the region of the telotroch (Fig. 1A,C,D). *BmiElav* thus includes the region of the future dorsal and ventral nerve cords. Higher magnification of the perianal field reveals additional scattered *BmiElav*+ cells laterally outside the nerve cords (Fig. 1E).

In juvenile *B. misakiensis*, *Elav*+ cells are abundant in all condensed parts of the nervous system^20^, including the proboscis plexus at the base of the proboscis region and the proboscis nerve ring (Fig. 1A’B’). At the level of the collar region, *BmiElav*+ cells locate to the subepidermal collar cord (Fig. 1D’). *BmiElav*+ cells are also present in the prebranchial nerve ring, as well as in the dorsal and ventral nerve cords in the trunk region (Fig. 1B’,C’). The *BmiElav* signal is interrupted in the dorsal nerve cord at the former position of the telotroch.

### Gene expression of anteroposterior patterning genes

We studied the expression of selected axial patterning genes to understand the interspecific variation of enteropneust collar cord development, and to compare with the expression pattern of the chordate neural tube.

The transcription factor *BmiSix3/6* is strongly expressed throughout the entire ectoderm of the proboscis region and extends into the anterior rim of the collar ectoderm in metamorphosing larvae (Fig. 2A,B) and juvenile worms (Fig. 2C,D).

*BmiOtx* is expressed in the metamorphosing larva in the ventral area of the proboscis nerve ring (Fig. 2F) and in a distinct annular domain, encircling the anterior and middle collar region (Fig. 2E,F, Fig. S1B). There is an additional domain in the anterior pharyngeal region, which is the developing stomochord (Fig. 2E arrowheads). This is a non-neural endodermal domain where the skeletal horns of the cartilaginous proboscis skeleton will later form. In the juvenile enteropneust *BmiOtx* is expressed in the ventral and ventrolateral area of the proboscis nerve ring (Fig. 2G,H). The expression forms a U-shaped domain at the position where the sensory pre-oral ciliary organ develops (Fig. 2G inset). *BmiOtx* is also weakly expressed throughout the ectoderm of the collar region (Fig. 2F,H).

*BmiEn(grailed)* is expressed in a circumferential ring at the very posterior margin of the collar region in metamorphosing larvae (Fig. 2I,J and Fig. S1C). The signal is ectodermal and interrupted at the level of the dorsal midline. The juvenile enteropneust shows a similar expression pattern at the posterior margin of the collar region (Fig. 2K,L). The ring of *BmiEn* expression shows a gap on the dorsal side, as in the metamorphosing larva.

In summary, the collar cord, which is part of the enteropneust collar region (mesosome), borders anteriorly the expression domain of *BmiSix3/6*, lies within the *BmiOtx*-expression region, and is posteriorly delineated by a line of *BmiEn* expression.

### Gene expression of mediolateral patterning genes

**Figure 3:**
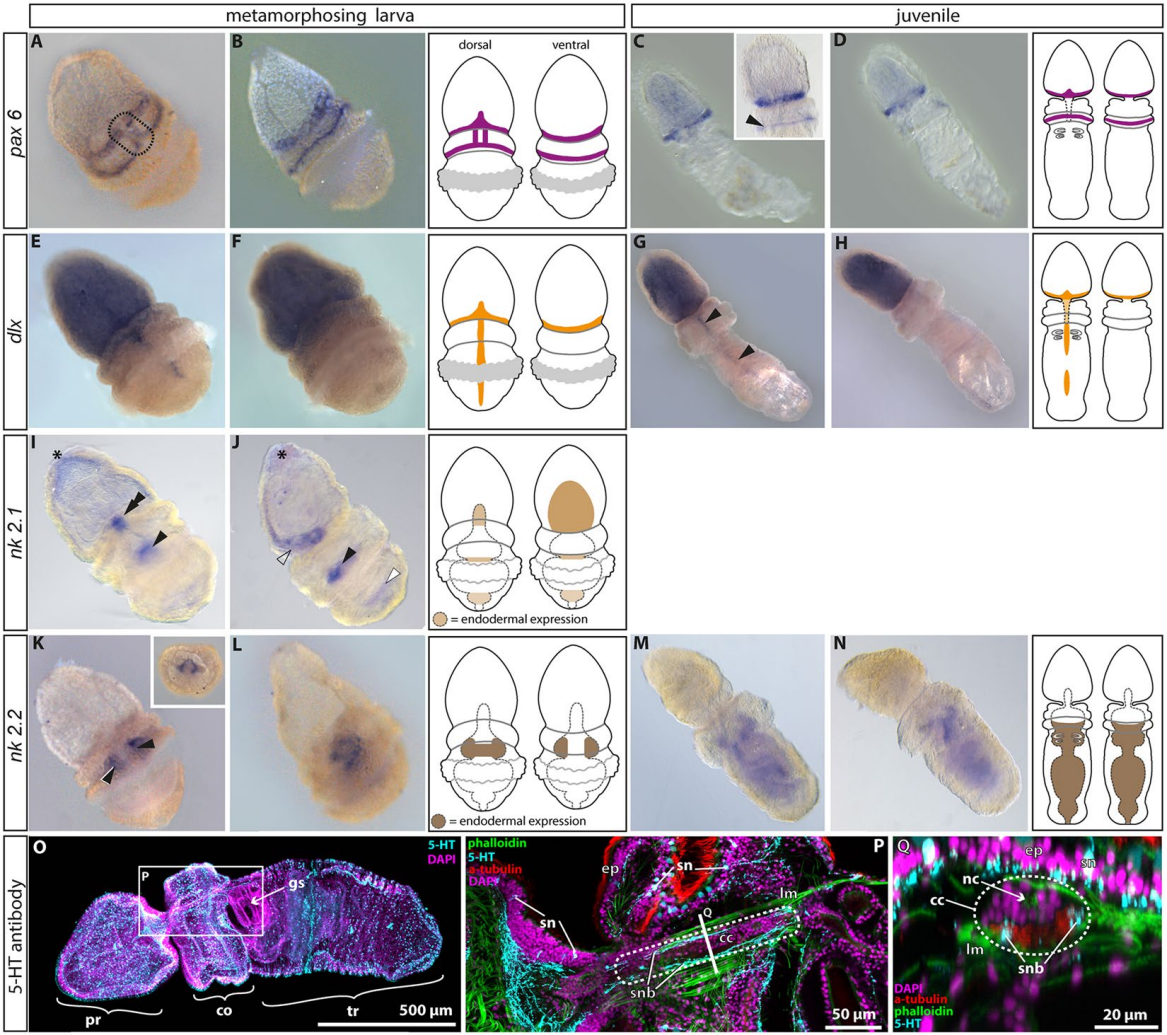
Expression domains of mediolateral patterning genes and serotonin-LIR in *B. misakiensis*. (**A,B**) *BmiPax6* is expressed in the proboscis nerve ring and in a second circumferential domain in the collar ectoderm. Additionally, *BmiPax6* forms paired longitudinal domains in the neural plate (dashed area) of the developing larva. In juveniles, the expression in the collar ectoderm fades (inset) and only the proboscis nerve ring shows strong signal of *BmiPax6* (**C,D**). (**E**–**H**) *BmiDlx* is expressed as a median stripe in the collar and dorsal cord (arrowheads) with an interruption at the level of the telotroch (**E**). The strong staining in the protocoel is unspecific (see also Fig. S1). (**I,J**) Expression of *BmiNk2.1* in the metamorphosing larva. *BmiNk2.1* is strongly expressed in the stomochord (double arrowhead), in the ventrolateral ectoderm at the base of the proboscis (open arrowhead), in the posterior pharynx (black arrowhead), and weakly in the hindgut (white arrowhead). The degrading apical organ shows a faint signal (asterisk). (**K**–**N**) Expression of *BmiNk2.2*. (**K**) Surface view from ventral showing bilateral domains in the mid-pharynx region. (**L**) Lateral view from left. Inset shows a cross section of the collar region with the ventrolateral domain of *BmiNk2.2* in the pharyngeal endoderm. In juveniles the expression domain of *Nk2.2* is extended throughout the entire endoderm (**M,N**). Note that there is no ectodermal or neuronal expression domain of *BmiNk2.2*. (**O**–**Q**) Serotonin-LIR in the juvenile. (**O**) Overview. (**P**) Detail of the collar region as indicated in G. Partial Z-projection focussing on the collar cord (dashed area). (**Q**) Virtual cross section of the collar cord as indicated in H. Note that 5-HT + somata are absent from the collar cord (dashed area). Only two ventrolateral 5-HT + neurite bundles pass the ventral area of neurites. cc = collar cord, co = collar, ep = epidermis, g s = sill slit, lm = longitudinal muscles, nc = neural canal, pr = proboscis, sn = serotonin-LIR neuron, snb = serotonin-LIR neurite bundle, tr = trunk. (**A,C,E,G,I,K,M**): dorsal views, (**B,J,L,N**): lateral views left, (**D,F,H**): ventral views.

In metamorphosing larvae, *BmiPax6* is strongly expressed in the proboscis nerve ring at the base of the proboscis and in an additional circular pattern in the ectoderm of the collar region (Fig. 3A,B). Between both circumferential domains, *BmiPax6* is also expressed in two parallel, longitudinal domains of the collar (Fig. 3A, dashed area). This area of the neural plate will later neurulate to form the subepidermal collar cord^13^. In juveniles, *BmiPax6* still shows a strong signal in the proboscis nerve ring. The circular domain in the posterior collar region becomes fainter in early juveniles (Fig. 3C inset) and is lost in older juveniles (Fig. 3C,D). No collar cord *BmiPax6* expression domains are present in juveniles.

Expression of *BmiDlx* is present in the proboscis nerve ring and along the dorsal nerve cord with an interruption at the level of the telotroch in the metamorphosing larva (Fig. 3E,F). In juveniles of *B. misakiensis, Dlx* expression shows a faint signal in the ventral and ventrolateral portion of the proboscis nerve ring and in the dorsal nerve cord including the collar cord (Fig. 3G,H). Our data show that *BmiDlx* is expressed in the collar cord and in the dorsal nerve cord and forms a single median domain.

*BmiNkx2.1* has four distinct expression domains in the metamorphosing larva (Fig. 3I,J). At this stage, the apical organ is degrading^20^ and *BmiNkx2.1* is weakly expressed in this ectodermal region (Fig. 3I,J asterisk). *BmiNkx2.1* shows a strong expression domain in the ventral ectoderm at the base of the proboscis region (Fig. 3J unfilled arrowhead). Further strong domains are within the developing endodermal stomochord (Fig. 3I, double arrowhead) and medially in the posterior pharyngeal endoderm (Fig. 3I,J black arrowhead). A fifth signal is present in the hindgut (Fig. 3J, white arrowhead).

The transcription factor *BmiNkx2.2* is strongly expressed in the lateral and dorsal portions of the anterior pharyngeal endoderm in the metamorphosing larva (Fig. 3K, inset, L). In the juvenile worm the *BmiNkx2.2* domain has extended posteriorly and is present throughout the endoderm, but absent from the hindgut (Fig. 3M,N). Thus, there is no expression domain of *Nk2* genes in the collar cord or the trunk nerve cords in *B. misakiensis*.

We additionally checked the distribution of serotonin-LIR neuronal components within the collar cord, because these neurons are restricted to the *Nkx2.1/2.2* domains in annelids and chordates. The serotonin-like immunoreactivity (LIR) nervous system of *B. misakiensis* has been previously described^20^, but the precise position of serotonin-LIR neurites within the collar cord has remained unknown. In the juvenile enteropneust serotonin-LIR neurons are present in the epidermis throughout all three body regions, with higher concentrations of somata in the proboscis and collar epidermis (Fig. 3O). Serotonin-LIR neurites form a basiepidermal nerve plexus in the proboscis and collar region. In the trunk region the serotonin-LIR neurites are condensed within the dorsal and ventral midline, in regions that constitute the nerve cords^20^. The neurulated collar cord is positioned between the dorsal mesenteries of the paired mesocoel and is composed of a dorsal sheath of cells and a ventral area of neurites (Fig. 3P,Q). The dorsal sheath of cells of the collar cord is devoid of serotonin-LIR somata (Fig. 3Q). Only two small ventrolateral serotonin-LIR neurite bundles pass through the whole neurite bundle of the collar cord. These serotonin-LIR lateral neurite bundles run adjacent to a pair of longitudinal muscle bundles, which run within the perihaemal diverticula that flank the collar cord ventrolaterally (Fig. 3Q).

## Discussion

We investigated the expression domains of several genes involved in axial as well as mediolateral patterning of the nervous system of the indirect developing enteropneust *Balanoglossus misakiensis*. By using the pan-neuronal marker *Elav* for differentiating neurons^22–24^, we found that the condensed parts of the adult nervous system (proboscis plexus and ring, neural plate, ventral and dorsal nerve cords) are already prepatterned by *Elav* in metamorphosing larvae prior to settlement. In settled juveniles of *B. misakiensis*, neurulation results in a subepidermal tubular cord, as reported in other enteropneust species^12, 13, 18^.

### Gene expression patterning of the collar cord in Enteropneusta

**Figure 4:**
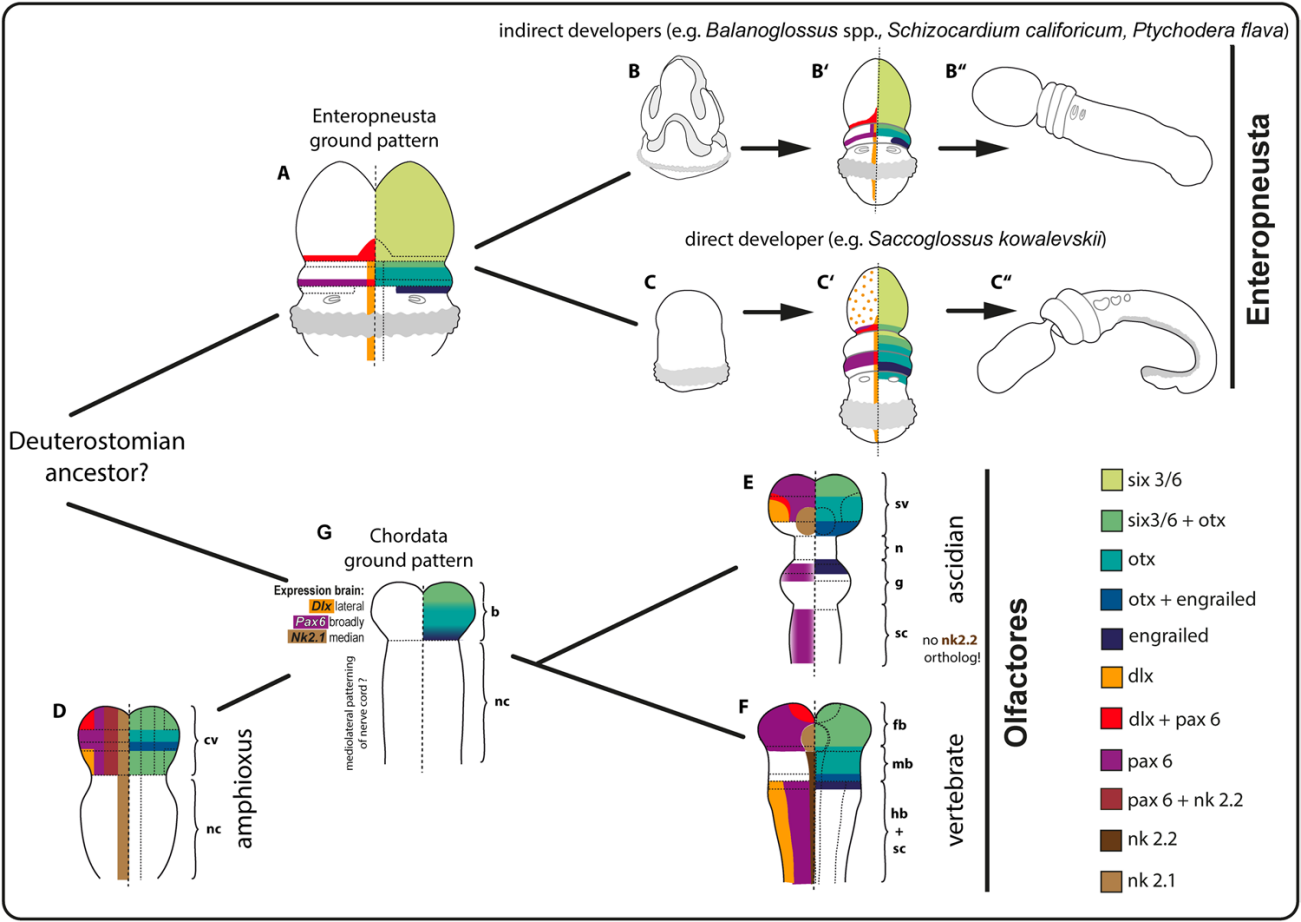
Comparison of axial patterning genes in the neural plate of diverse deuterostomian taxa with focus on different developing modes in enteropneusts. Neuronal patterning in enteropneusts is highly conserved and independent of the mode of development. The ancestral condition of mediolateral patterning for Deuterostomia remains elusive. See text for discussion. (**A**) Expression domains of the hypothetical enteropneust ancestor. (**B–B”**) Selected developmental stages of *B. misakiensis*. (**B**) Metschnikoff larval stage. (**B’**) Metamorphosing Agassiz larval stage (this study). (**B”**) Juvenile worm. (**C–C”**) Selected developmental stages of *S. kowalevskii*. (**C**) Torpedo embryo stage. (**C’**) 1-gill slit hatchling (after data from refs 15, 16). (**C”**) Juvenile worm. (**D**) Expression domains in the neural plate of *Branchiostoma floridae* (after data from refs 34–36, 38, 39, 55). (**E**) Expression domains in the neural plate of the ascidian *Ciona intestinalis* (after data from refs 34, 40, 56–58). F Expression domains in the neural plate of the vertebrate *Mus musculus*. Scheme modified after^2^ (after data from refs 3, 16, 31, 37). (**G**) Expression domains of the hypothetical chordate ancestor. Mediolateral patterning of the postcerebral nerve cord is ambiguous. Note, all expression patterns are symmetrical, but are shown on one side only for clarity. b = brain, cv = cerebral vesicle, fb = forebrain, g = ganglion, hb = hindbrain, mb = midbrain, n = neck, nc = nerve cord, sc = spinal cord, sv = sensory vesicle.

The transcription factors *Six3/6*, *Otx* and *Engrailed* play a conserved role in anteroposterior patterning and regionalization of the nervous system in chordates and in many other bilaterians^3^. *Six3/6* patterns the anteromost region of the nervous system in numerous animals^3, 25, 26^. We found that in *B. misakiensis* the expression pattern of *Six3/6* is likewise at the anteriormost region of the animal, while *Otx* and *Engrailed* form circular epidermal domains around the collar and the posterior margin of the collar region, respectively (Fig. 4B’). These expression domains are spatially similar to what has been described in the spengelid *Schizocardium californicum* ^17^ as well as the harrimaniid enteropneust *Saccoglossus kowalevskii* (Fig. 4C’^15, 16^,) Accordingly, we suggest a conserved role of neuronal and body region patterning for *Six3/6*, *Otx* and *Engrailed* in Enteropneusta that is independent from their mode of development (direct vs. indirect, Fig. 4B–C”). It is a plesiomorphic feature for Enteropneusta that has been inherited from a common bilaterian ancestor^3, 15, 25^.

Next, we examined the expression pattern of *Dlx*, *Pax6* and *Nk2.1/2.2*. These transcription factors form mediolateral neurogenic domains in the neural tube of mouse, fruit fly and in the annelid *Platynereis dumerilii* ^19, 27^. Our analysis of *B. misakiensis* shows that *BmiDlx* is expressed in a narrow longitudinal stripe in the dorsal midline of the neural plate (Fig. 4B’). A similar pattern has been reported for *Dlx* in *S. kowalevskii* (Fig. 4C’^16, 28^,), *S. californicum* ^17^ and *Balanoglossus simodensis* ^18^, suggesting a conserved role of this transcription factor in neurogenesis in Enteropneusta. We then examined the expression pattern of *BmiPax6* and found that it forms two lateral stripes along the neural plate of *B. misakiensis* (Fig. 4B’). We show that *BmiPax6* is only expressed for a short period in the neural plate during metamorphosis and is entirely absent in early juveniles (2 d post-settlement) (Fig. 3C,D). This is the only report of a distinct expression pattern of *Pax6* in the neural plate of an enteropneust species. In a comparable developmental stage of *S. kowalevskii* (1-gill-slit stage), *Pax6* is expressed in corresponding circular domains (Fig. 4C’^15, 16^,), yet details from the neural plate are unknown. In *B. simodensis* and *S. californicum*, *Pax6* expression was not detected in the neural plate^17, 18^. Thus, *Pax6* expression in the collar cord might be a species-specific acquisition of *B. misakiensis* and not part of the enteropneust ground pattern (Fig. 4A).

Expression pattern analysis of the median progenitor markers *Nk2.1* and *Nk2.2* revealed that there are no expression domains for either of the *BmiNk2* genes in the developing neural plate nor, later, in the collar cord in *B. misakiensis*. Instead, the main domains of *Nk2.1* and *Nk2.2* are detected in the pharyngeal endoderm (Fig. 3I–N). In the direct developer *S. kowalevskii* a similar endodermal expression of both genes has been reported previously^15, 28^ and in adult *Ptychodera flava Nk2.1* also shows similar expression domains^29^, suggesting a more general role in endoderm specification of these genes in enteropneusts^30^. Moreover, serotonergic neurons in vertebrates are usually restricted to the progenitor domains of *Nk2.1/2.2* ^31^. Our data show that there is no median *Nk2.2* domain in the collar cord in *B. misakiensis*. Concordantly, no serotonin-LIR somata are present in the collar cord of *B. misakiensis*. In fact, *Nk2.2* does not co-localise with serotonin-LIR neurons in *B. misakiensis* Serotonin-LIR neurons comprise bipolar neurons throughout the epidermis of *B. misakiensis*, *S. kowalevskii* ^20^ as well as *P. flava* ^14^.

Concluding so far, with the exception of *Pax6*, the expression patterns of the genes investigated in this study are highly congruent among the enteropneusts *S. kowalevskii, S. californicum, B. simodensis, P. flava* as well as *B. misakiensis*, and thus similar functions appear most likely. The data further reveals that neuronal patterning of the adult nervous system in the different families of Enteropneusta (Harrimaniidae, Spengelidae and Ptychoderidae) is not affected by different developmental modes. This conclusion is also supported by morphogenetic data of the developing adult nervous system in enteropneusts^20^. On this basis, we propose that a similar collar cord patterning was present in the last common ancestor of Enteropneusta (Fig. 4A).

### Comparative aspects of neural tube patterning among deuterostomes

Morphological similarities between the tubular collar cord and the chordate neural tube have not gone unnoticed, dating back more than 130 years^32^. Therefore, we compare the gene expression patterns of the transcription factors studied here among different deuterostomes, and discuss the evolutionary implications.

Chordata comprises three major taxa, Cephalochordata, Tunicata and Vertebrata, of which the latter two form the monophyletic Olfactores^7, 33^. All three groups share corresponding expression domains of the transcription factors *Six3/6, Otx* and *Engrailed* (Fig. 4D–F), which are restricted to the anterior portion of the neural plate, i.e., the future brain region^3, 34^. Thereby, coexpression of *Otx* and *En* mark the future midbrain-hindbrain boundary (MHB) in vertebrates and the posterior margin of the future sensory vesicle (brain) in the ascidian *Ciona intestinalis*. In contrast, the coexpressing domain of *Otx* and *En* in amphioxus is located in the midlevel of the brain region, whereas a second expression domain of *Six3/6* is present at the posterior end of the cerebral vesicle (Fig. 4D)^3, 35^. Moreover, all three groups show a median/ventral *Nk2.1* domain and expression domains of *Pax6* and *Dlx* in the brain region^15, 19, 31, 36–40^. Thus, the chordate ancestor likely had a similar brain patterned by these transcription factors (Fig. 4G)^3^.

Mediolateral patterning of the postcerebral part of the neural tube by *Pax6*, *Dlx* and *Nk2.1/2.2* differs considerably between chordates and needs further attention. The specific arrangement of lateral *Dlx*, mediolateral *Pax6* and median *Nk2* domains has been reported from the vertebrate spinal cord and hindbrain levels (posterior to MHB) as well as from the annelid and insect ventral nerve cord (postcerebral)^2, 3^. The median column of *Nk2.2* is an exception as its domain projects anteriorly throughout the midbrain region and is replaced by *Nk2.1* in the vertebrate forebrain (Fig. 4F). However, ascidians share only a mediolateral *Pax6* domain with vertebrates, while *Dlx* and *Nk2.2* expression is absent from the postcerebral neural tube (Fig. 4E)^3, 40, 41^. Ascidians belong to Tunicata, a taxon of rapidly evolving animals with reduced genome size that have lost about 25 genes involved in developmental patterning including *Gbx*, *Wnt1* and *Nk2.2* ^34, 42, 43^. Thus, the aberrant and missing expression domains as compared to vertebrates could be explained by secondary gene losses in Tunicata. In comparison, amphioxus does not appear to be rapidly evolving. Cephalochordates have retained all of the putative ancestral bilaterian homeobox genes^34, 43^ and amphioxus exhibits one of the most ancestral genomes among chordates, in parallel with a less derived morphology^43^. However, *Pax6* and *Dlx* expression are absent from the nerve cord in amphioxus, instead the median *Nk2.1* domain extends throughout the posterior neural plate (Fig. 4D)^39^. It should be mentioned that a median *Nk2.1/2.2* domain, a mediolateral *Pax6* as well as a lateral *Dlx* expression domain are very well present in amphioxus, yet these expression domains are located in the posterior region of the cerebral vesicle (Fig. 4D) and not in the postcerebral nervous system as in vertebrates (Fig. 4D,F) and the protostomes *Platynereis dumerilii* and *Drosophila melanogaster* ^2, 19^. Taken together, mediolateral expression domains of *Pax6*, *Dlx* and *Nk2* genes in the postcerebral nerve cord differ considerably among chordates, making it difficult to suggest a complete ancestral ground pattern for Chordata (Fig. 4G).

The reconstructed enteropneust ground pattern (Fig. 4A) allows for a comparison of tubular nervous systems among deuterostomes and will contribute to understanding their evolution. In this context, echinoderms are not considered, because they possess a highly derived body plan (pentamery, oral-aboral axis) and ancestrally a non-tubular nervous system^44^.

Comparison of the expression domains of *Six3/6*, *Otx* and *Engrailed* leads to the suggestion that the collar cord in enteropneusts might correspond to a region of the chordate brain rather than to the postcerebral neural tube (Fig. 4A,G). This is also supported by comparative *Hox* gene expression in *S. kowalevskii* ^3, 15^. In Enteropneusta the neural plate is patterned medially by *Dlx* (refs 15, 16 and 18, this study) (Fig. 4A), whereas *Dlx* expression is restricted to the very lateral area of the brain in amphioxus and ascidians (Fig. 4D,E) with respect to the spinal cord in vertebrates (Fig. 4F). Accordingly, there is no corresponding mediolateral patterning present in the enteropneust nervous system, and compared to chordates the expression domains of *Dlx* and *Pax6* are flipped in *B. misakiensis* (Fig. 4B’,D–F). These incongruent expression patterns might be explained by the fact that dorsoventral expression of *Bmp* and *Chordin*, which are responsible for the placement of the mediolateral patterning domains, are reversed in enteropneusts relative to chordates^28^. In *S. kowalevskii* the tubular collar cord develops from the *Bmp*-expressing side, whereas the dorsal neural tube of chordates and the ventral nerve cord of protostomes form at the *Chordin*-expressing side^28, 45^. Concordantly, markers of midline cells in the chordate neural tube such as *Sim* and *Netrin* are expressed in the ventral ectoderm in enteropneusts while lateral markers of the chordate neural tube such as *Dlx* are expressed in the dorsal ectoderm (ref. 28, this study). Thus, following the dorsoventral (D-V) inversion hypothesis^46, 47^ (but see ref. 48), the *dorsal* side of chordates (neural tube) corresponds to the *ventral* side of enteropneusts, yet the collar cord is positioned dorsally. Accordingly, the enteropneust collar cord and the chordate neural tube do not represent corresponding parts of related organisms, making a homology hypothesis debatable.

## Conclusion

A complex mediolateral patterning of the postcerebral nervous system by *Pax6*, *Dlx* and *Nk2.1/Nk2.2* has been reported in vertebrates and the protostomes *Platynereis dumerilii* and *Drosophila melanogaster* ^2, 3, 19^. However, comparison of their expression domains between enteropneusts and chordates (amphioxus, ascidians and vertebrates) does not suggest that a similarly patterned postcerebral nervous system was present in the last deuterostomian ancestor (Fig. 4). Moreover, the tubular collar cord of Enteropneusta shows no expression domains of *Dlx* or *Pax6* that correspond to the chordate brain. The “flipped” domains of *Dlx* and *Pax6* in enteropneusts are likely the result of an inverted *BMP/Chordin* expression compared to chordates^28^. Previous electrophysiological and morphological data also proposed limited resemblances between the collar cord and neural tube^10, 49^. Altogether, it should be considered that the collar cord might represent an independent acquisition of Enteropneusta and that tubular nervous systems evolved convergently within Deuterostomia.

## Materials and Methods

### Balanoglossus misakiensis (Kuwano, 1902)

Adult *B. misakiensis* were collected at a depth of 1 to 2 m at Sunset beach, Aomori-Bay, Asamushi, Aomori, Japan, in June 2012 and June 2014. Specimens were transported to the Research Center for Marine Biology Tohoku University in Asamushi and were kept in aquaria with running filtered seawater at ambient water temperature (24–26 °C) as previously described^20, 50^. Spawning, *in vitro* fertilization, and fixations were performed as described earlier^20^.

### Immunolabelling and confocal laser scanning microscopy

Juveniles of *B. misakiensis* (2-gill-slit juvenile = 3 days post settlement) were fixed with 4% paraformaldehyde (PFA) in phosphate buffer (PBS). Specimens were processed using standard protocols as previously described^20^.

### RNA extraction, transcriptome analysis and gene cloning

More than 1,000 larvae from developmental stages of *B. misakiensis* ranging from early hatched tornaria to three day old juvenile worms were fixed in RNAlater (Sigma). Total RNA was extracted from a mix of developmental stages using RNeasy Mini Kit from Qiagen. Extracted RNA was sent to Eurofins (Germany) for Illumina HiSeq. 2000 sequencing using paired-end read module resulting in reads of 100 bp length. Obtained reads were assembled to contigs using Trinity v2.0.1 software under standard parameters and the transcriptome was analysed for sequences of interest with sequence search in Geneious 6.1 (Biomatters, New Zealand). Primers were generated with Primer3 software to obtain fragments of *Elav, Six3/6, Pax6, Dlx, Otx, Engrailed, Nk2.1* and *Nk2.2* (for primer sequences and accession numbers see supplemental material), synthesized by Microsynth AG (Balgach, Switzerland) in order to sub-clone into pGemT Easy vector (Promega).

### Phylogenetic analysis

Full protein sequences were aligned using MUSCLE and regions with low-quality alignments for the Elav phylogenetic analysis were trimmed by TrimAl 1.2 rev 59 using the option “automated1” for the trimming^51^. For the reconstruction of the phylogenetic relationship between analysed homeobox proteins, the alignment was not trimmed. ProtTest 2.4^52^ analysis retrieved LG (+G+F) and JTT (+I+G+F) as best-fitting models for the amino substitution rates for Elav (Fig. S2) and the homeobox protein analyses (Fig. S3), respectively. The maximum likelihood trees of protein sequences were then generated with PhyML 3.0^53^ with default parameters and selecting the suitable amino acid substitution models previously identified. The tree topology search operations were set to the nearest neighbor interchange and subtree pruning and regrafting methods to retrieve the best solution for the estimation of the tree topology. The support values were calculated using 100 non-parametric bootstrap replicates.

### Probe synthesis and *in situ* hybridization

Chromogenic *in situ* hybridizations were performed on whole-mounts with a pierced protocoel (see Controls for more information) following the protocol from Röttinger and Martindale^54^ with minor adjustments for *B. misakiensis*. Metamorphosing larvae (Agassiz stage) and juveniles (2-gill-slit stage) were treated with 10 ng/µl Proteinase K (Roth) for 4 min at room temperature. Colour development was stopped by three washes in PTw (phosphate buffered saline + 0.1% Tween20) and postfixed with 4% PFA for 1 hour. Animals were transferred into 100% EtOH over night for clearing and mounted in 80% glycerol.

### Controls

Controls with sense probes were conducted in order to identify unspecific binding and probe trapping during *in situ* hybridization. The protocoel within the proboscis region turned out to be a perfect trap for any probe (Fig. S1A). Perforation of the proboscis using a thin tungsten needle helped to solve this problem (Fig. S1D,E). However, probe trapping could not always be eliminated, which is why a blue protocoel persists in the *in situ* hybridizations of *Dlx* (Fig. 3E–H), *Otx* and *Engrailed* in juveniles (Fig. 2G,H,K,L).

## Electronic supplementary material

Supplementary Information
