# Supplementary material for: Neuronal patterning of the tubular collar cord is highly conserved among enteropneusts but dissimilar to the chordate neural tube
Source: Sci Rep. 2017 Aug 1;7:7003. doi: 10.1038/s41598-017-07052-8 (PMC5539250; doi:10.1038/s41598-017-07052-8)
Supplement: Supplementary file 1 — Supplementary Information [file 41598_2017_7052_MOESM1_ESM.pdf]

Neuronal patterning of the tubular collar cord is highly conserved among enteropneusts but dissimilar to the chordate neural tube

Sabrina Kaul-Strehlow, Makoto Urata, Daniela Praher, Andreas Wanninger

Supplementary figure 1

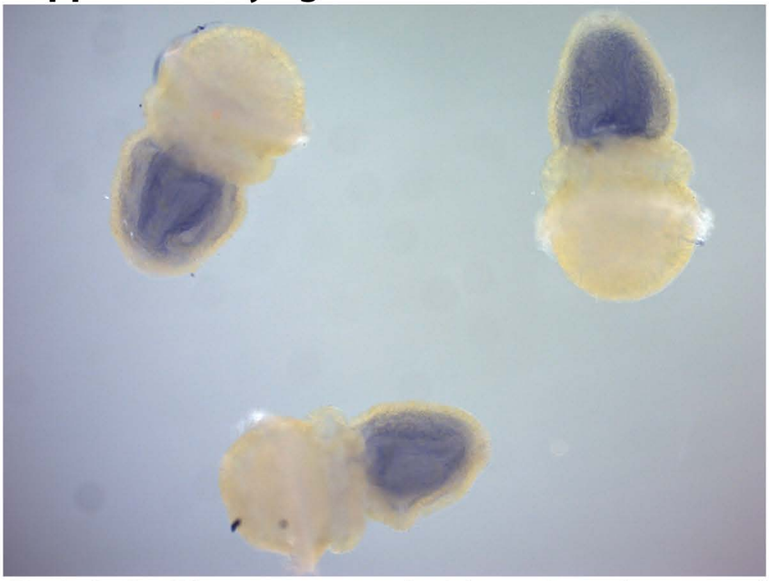

Fig.S1A: *BmiElav* sense control with unpierced protocoel showing probe trapping in the protocoel.

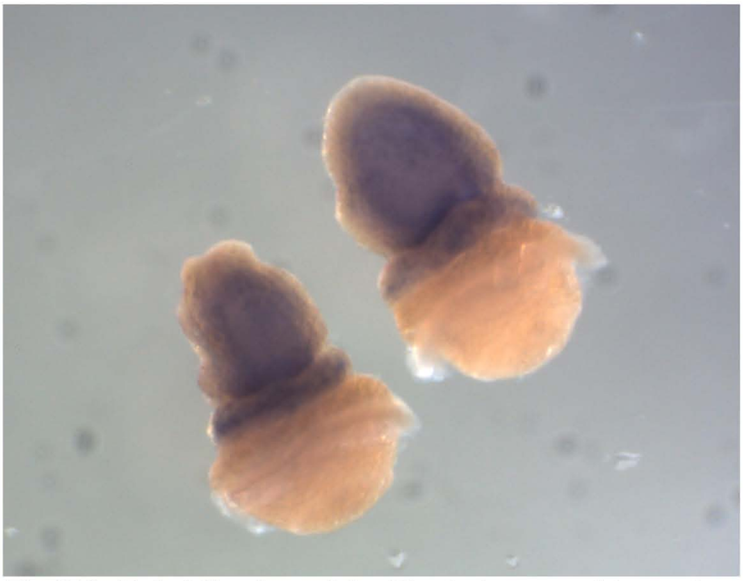

Fig.S1B: Hybridization of *BmiOtx* in larvae with unpierced protocoel showing probe trapping in the protocoel.

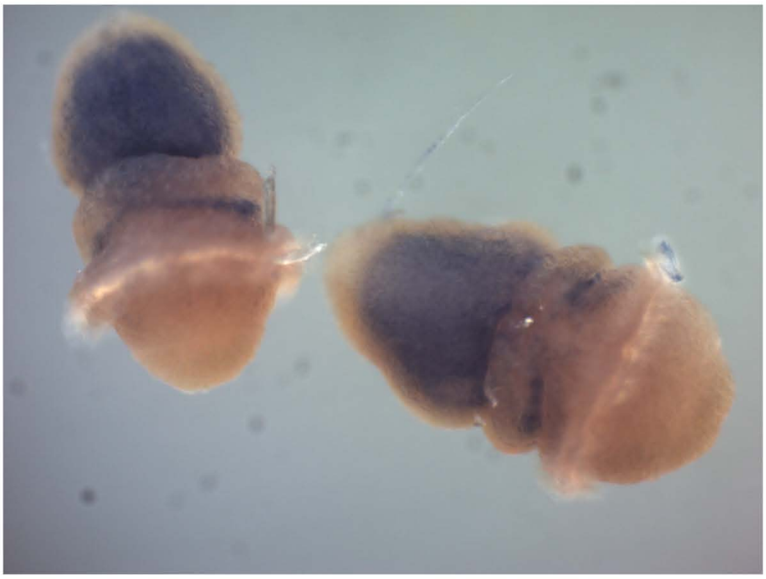

Fig.S1C: Hybridization of *BmiEn* in larvae with unpierced protocoel showing probe trapping in the protocoel.

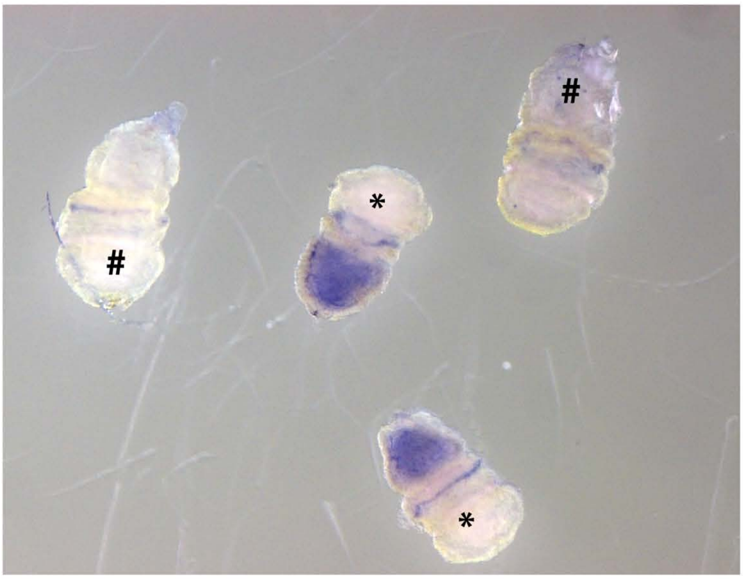

Fig.S1D: Hybridization of *BmiEn* in larvae with unpierced (*) AND pierced (#) protocoel for comparison.

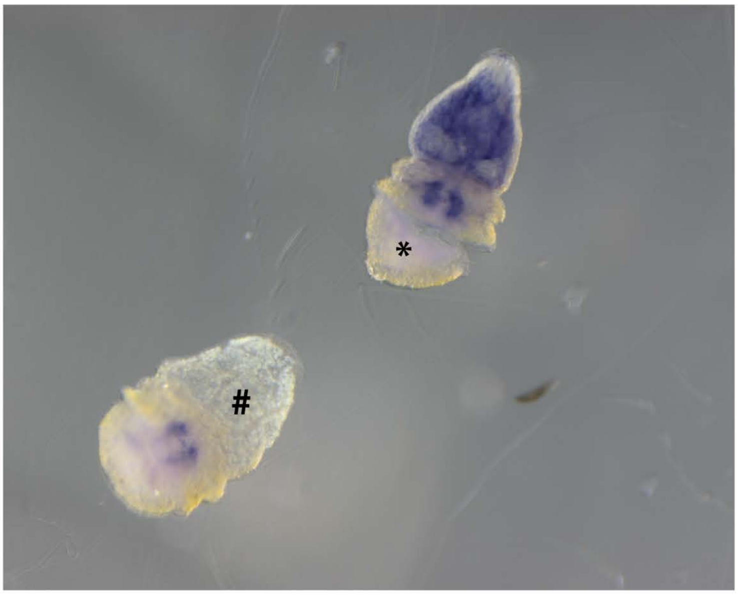

Fig.S1E: Hybridization of *BmiNk2.2* in larva with unpierced (*) AND pierced (#) protocoel for comparison.

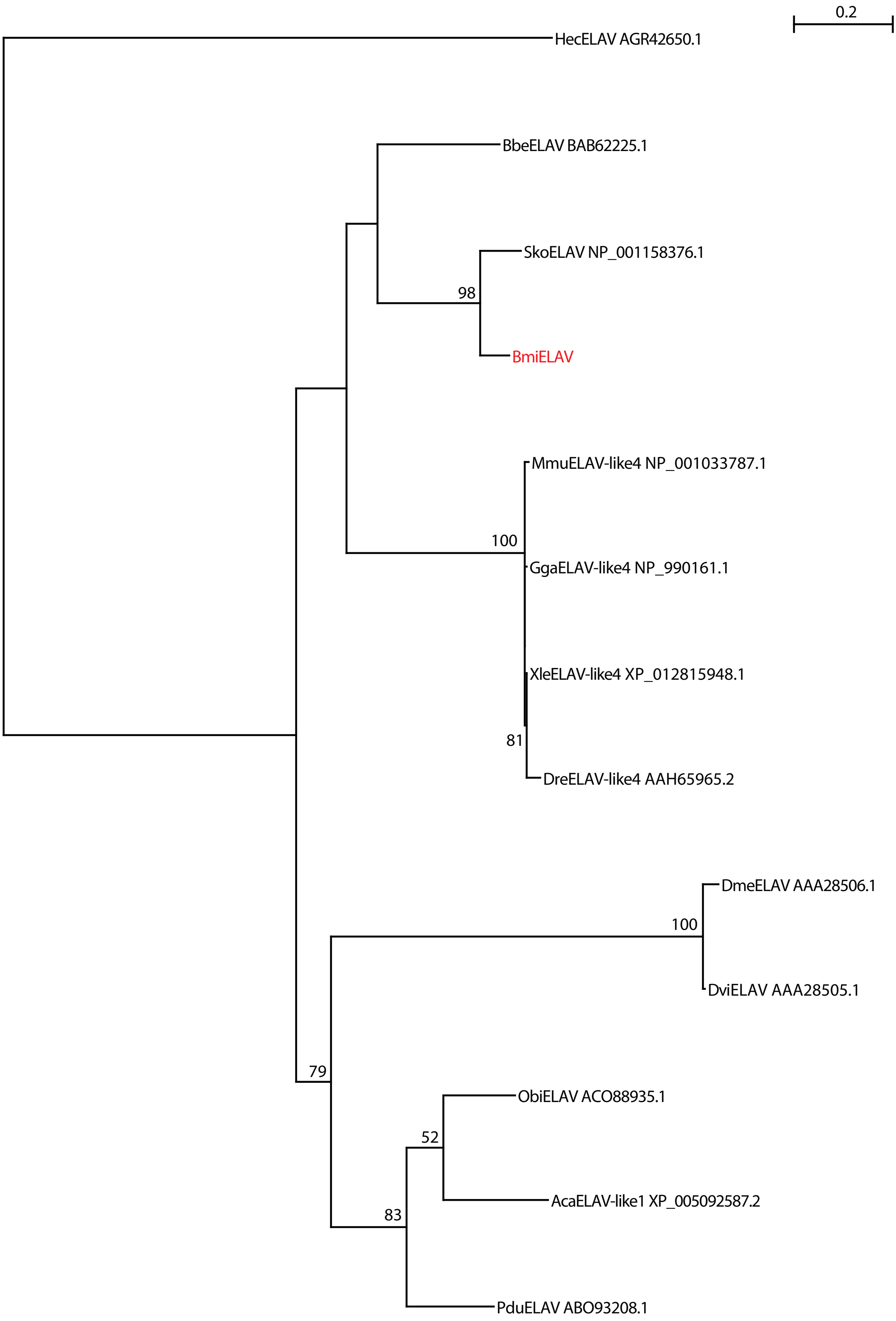
0.2
HecELAV AGR42650.1
BbeELAV BAB62225.1
SkoELAV NP_001158376.1
98
BmiELAV
MmuELAV-like4 NP_001033787.1
100
GgaELAV-like4 NP_990161.1
XleELAV-like4 XP_012815948.1
81
DreELAV-like4 AAH65965.2
DmeELAV AAA28506.1
100
DviELAV AAA28505.1
79
ObiELAV ACO88935.1
52
AcaELAV-like1 XP_005092587.2
83
PduELAV ABO93208.1

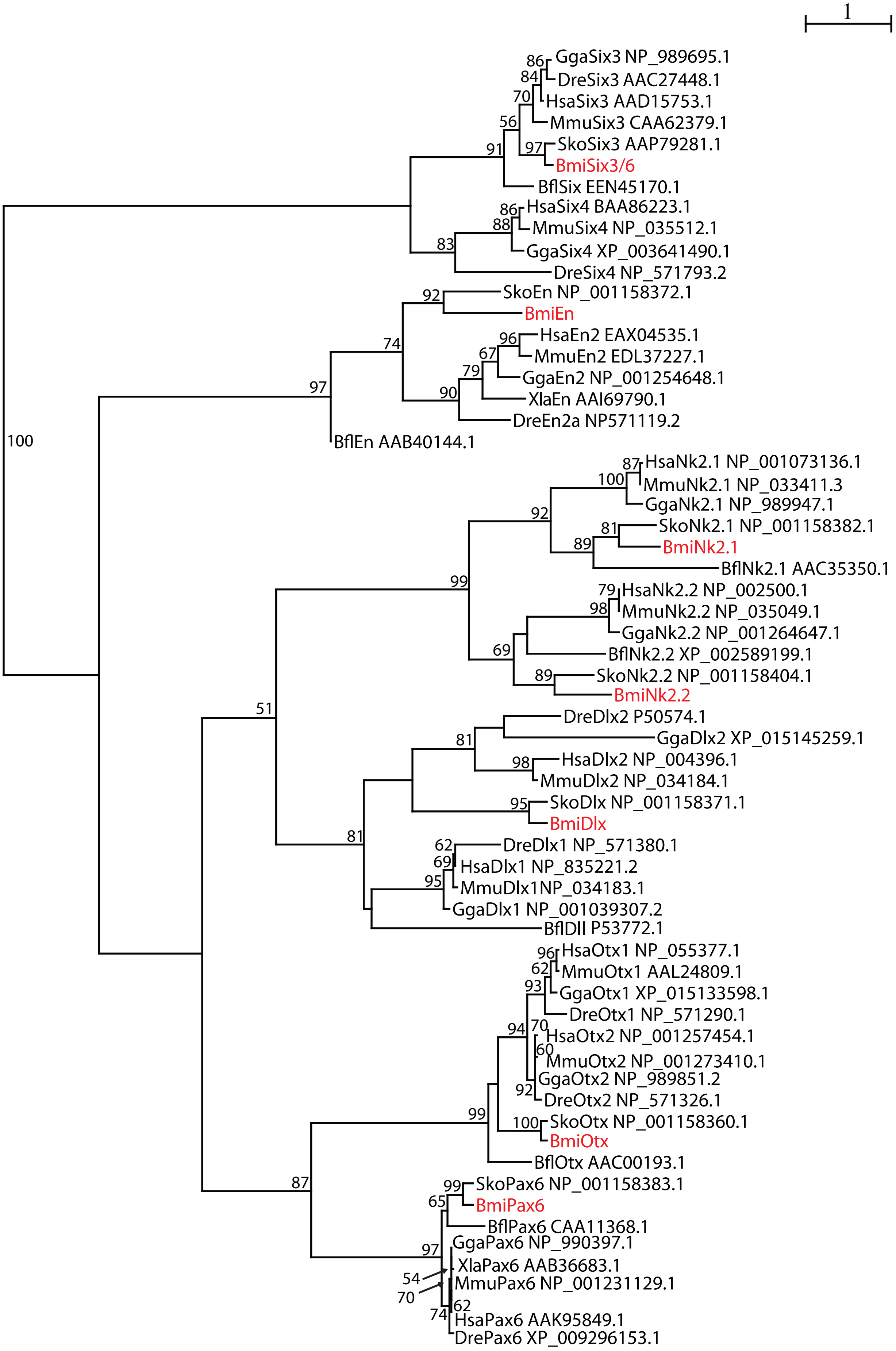
1
GgaSix3 NP_989695.1
DreSix3 AAC27448.1
HsaSix3 AAD15753.1
MmuSix3 CAA62379.1
SkoSix3 AAP79281.1
BmiSix3/6
BflSix EEN45170.1
HsaSix4 BAA86223.1
MmuSix4 NP_035512.1
GgaSix4 XP_003641490.1
DreSix4 NP_571793.2
SkoEn NP_001158372.1
BmiEn
HsaEn2 EAX04535.1
MmuEn2 EDL37227.1
GgaEn2 NP_001254648.1
XlaEn AAI69790.1
DreEn2a NP571119.2
BflEn AAB40144.1
HsaNk2.1 NP_001073136.1
MmuNk2.1 NP_033411.3
GgaNk2.1 NP_989947.1
SkoNk2.1 NP_001158382.1
BmiNk2.1
BflNk2.1 AAC35350.1
HsaNk2.2 NP_002500.1
MmuNk2.2 NP_035049.1
GgaNk2.2 NP_001264647.1
BflNk2.2 XP_002589199.1
SkoNk2.2 NP_001158404.1
BmiNk2.2
DreDlx2 P50574.1
GgaDlx2 XP_015145259.1
HsaDlx2 NP_004396.1
MmuDlx2 NP_034184.1
SkoDlx NP_001158371.1
BmiDlx
DreDlx1 NP_571380.1
HsaDlx1 NP_835221.2
MmuDlx1NP_034183.1
GgaDlx1 NP_001039307.2
BflDll P53772.1
HsaOtx1 NP_055377.1
MmuOtx1 AAL24809.1
GgaOtx1 XP_015133598.1
DreOtx1 NP_571290.1
HsaOtx2 NP_001257454.1
MmuOtx2 NP_001273410.1
GgaOtx2 NP_989851.2
DreOtx2 NP_571326.1
SkoOtx NP_001158360.1
BmiOtx
BflOtx AAC00193.1
SkoPax6 NP_001158383.1
BmiPax6
BflPax6 CAA11368.1
GgaPax6 NP_990397.1
XlaPax6 AAB36683.1
MmuPax6 NP_001231129.1
HsaPax6 AAK95849.1
DrePax6 XP_009296153.1
100
91
56
70
84
86
97
83
88
86
97
74
92
90
79
67
96
51
99
92
100
87
89
81
69
98
79
89
81
81
98
95
95
69
62
87
99
94
93
62
96
70
60
92
100
97
65
99
54
70
74
62
